# Research on the Efficacy of Ganpu Vine Tea in Inhibiting Uric Acid Production

**DOI:** 10.3390/metabo13060704

**Published:** 2023-05-29

**Authors:** Zhi-Xu Zhang, Run-Ming Mo, Dong-Bo Liu, Yi-Song Liu, Cong-Hui Liu, Yong-Shen Li, Zhong-Hua Liu, Dan Qin

**Affiliations:** 1 Hunan Co-Innovation Center for Utilization of Botanical Functional Ingredients, Changsha 410128, China; chinasaga@163.com (D.-B.L.); liuyisong@hunau.edu.cn (Y.-S.L.); larkin-liu@163.com (Z.-H.L.); qd730101@163.com (D.Q.); 2 Horticulture College, Hunan Agricultural University, Changsha 410128, China; 3 National Research Center of Engineering Technology for Utilization Ingredients from Botanicals, Changsha 410128, China; 4 State Key Laboratory of Subhealth Intervention Technology, Changsha 410128, China; 5 College of Food Science and Technology, Hunan Agricultural University, Changsha 410128, China; 6 College of Veterinary Medicine, Hunan Agricultural University, Changsha 410128, China; 7 Hunan Kangqi 100 Biotechnology Ltd., Zhuzhou 412205, China; jializhou2021@163.com (C.-H.L.); cuiyarong1015@126.com (Y.-S.L.)

**Keywords:** Ganpu vine tea, xanthine oxidase, uric acid, in vitro enzyme inhibition model, cellular model

## Abstract

Ganpu vine tea is a new type of health care citrus fruit tea made from citrus shell, Pu-er tea, and vine tea baked as raw materials. In this study, the in vitro uric acid synthase inhibition system and hyperuric acid cell model were constructed to appraise the uric acid lowering efficacy of Ganpu vine tea, traditional Ganpu tea, and vine tea. Results showed that in the uric acid synthase inhibition system, the aqueous extract can inhibite the puric metabolically related enzymes, such as adenosine deaminase (ADA), purine nucleoside phosphorylase (PNP), and xanthine oxidase (XOD). The ability of the aqueous extract to inhibit the above enzyme was as follows: vine tea > Ganpu vine tea > Ganpu tea; all teas had a strong effect on XOD inhibition. The hyperuric acid cell model test showed that the aqueous extract inhibited uric acid production through accumulating inosine and hypoxanthine and hindering xanthine synthesis. The uric acid reductive ability was as follows: Vine tea > Ganpu vine tea > Ganpu tea. The inhibition of enzymes related to uric acid synthesis and the inhibition of uric acid production were significantly enhanced through adding vine tea to Ganpu tea. It also shows that flavonoids are the main factor driving this ability because they are the main active ingredients in these botanical drinks.

## 1. Introduction

**Figure 1:**
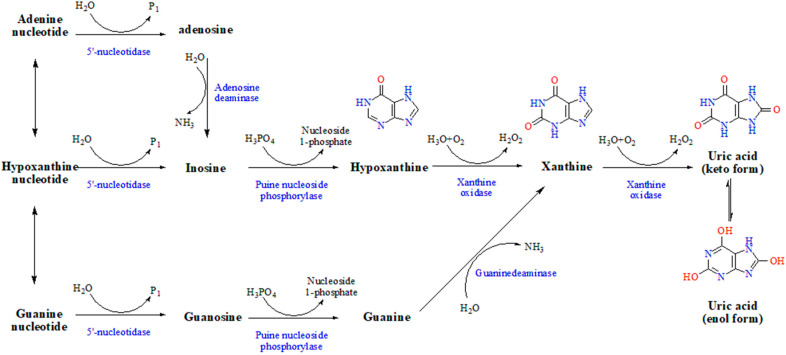
Uric acid synthesis pathway.

Uric acid is the end product of purine metabolism in the human body. Under normal conditions, the synthesis and decomposition of purine and the production and excretion of uric acid are in a relative balance in the human body. When a person consumes high-purine foods, a large amount of nucleic acid is broken down in the body due to tumors, or uric acid excretion is impaired due to renal disease, the concentration of uric acid in the blood exceeds the average level and develops hyperuricemia (HUA), which will lead to gout [1,2,3]. Past studies showed that HUA acts as an independent risk factor for various metabolic and cardiovascular diseases and can trigger conditions such as diabetes, hypertension, and atherosclerosis [4,5,6]. As shown in Figure 1, the xanthine pathway is the main pathway of uric acid production, while allopurinol, febuxostat, and topiramate all inhibit the cascade conversion of hypoxanthine to uric acid through inhibiting XOD (xanthine oxidase) activity, thereby reducing blood uric acid levels [4,7,8], XOD inhibitors are the traditional drugs of choice for combating HUA and gout [9]. However, drugs that inhibit uric acid production are associated with several side effects, including causing hypersensitivity reactions, liver and kidney damage, bone marrow suppression, and heart failure [9,10,11]. Past studies found that natural active ingredients in tea products (such as tea polyphenols and tea pigments) are effective in the prevention and treatment of HUA and gout. With multiple targets of function and few toxic side effects, they attracted widespread attention in the pharmaceutical and food fields [12,13,14].

Vine tea, which is also known as frosted tea, and has the scientific name is *Ampelopsis grossedentata*, is mainly distributed to the south of the Yangtze River. As a flavonoid-rich and caffeine-free tea, dihydromyricetin is the main flavonoid compound in vine tea, being above 43% and having anti-inflammatory, hypolipidemic, hepatoprotective and uric acid-reductive properties [15,16,17]. Past studies showed that vine tea can reduce serum uric acid value through decreasing liver XOD activity in mice and has a significant protective effect on liver and kidney tissue [18,19]. As a natural health tea, vine tea is mainly consumed via direct infusion, which is characteristic of freshness and sweetness. As it has the problems of heavy greenness, bitterness, and dark soup color, the development of the vine tea industry is limited. Blending different traditional teas with vine tea can reduce the undesirable flavor of vine tea; thus, various products, such as vine tea drinks, vine tea tablets, and vine tea jelly, were previously developed [20,21,22]. However, the application of vine tea in citrus fruit tea is rarely reported.

Citrus fruit tea is a type of tea–fruit composite reprocessed tea product made through hollowing out the flesh of fresh citrus fruit and filling it with tea leaves, drying and shrinking it through sunlight or drying, and making it via natural aging. The well-known Ganpu tea on the market is created through filling the citrus shell with Pu’er tea; the main chemical components are compared in Table 1 [23]. In this study, vine tea was innovatively blended with Ganpu tea to obtain a better flavor and enhanced health benefits. The study constructed an in vitro enzyme reaction system and high uric acid cell model, finally evaluating the in vitro enzyme activity inhibition and uric acid production inhibition of the optimized Ganpu vine tea, Ganpu tea, and vine tea to provide strong support for the development of citrus fruit tea with health care functions.

## 2. Materials and Methods

### 2.1. Main Reagents

Human hepatocellular carcinoma cell line (HepG2) was purchased from Shanghai Institute of Cell Science, Chinese Academy of Sciences. Adenosine, inosine, hypoxanthine, xanthine, uric acid, febuxostat (HPLC grade, purity ≥ 99%), XOD (xanthine oxidase, 10.9 U/mL), PNP (purine nucleoside phosphorylase, 59.4 U/mg), and ADA (adenosine deaminase, 16 U/mg) was purchased from Shanghai Yuanye Biological; sodium n-pentanesulfonate monohydrate (ion-pair chromatography grade, purity ≥ 99%) was purchased from Shanghai Maclean. Glacial acetic acid (excellent purity, purity (≥99.8%), potassium acetate (analytical purity, purity ≥ 92%), and methanol (HPLC grade, purity ≥ 99.8%) were purchased from Sinopharm; RMPI1640 medium, fetal bovine serum, phosphate buffer solution (PBS), and 0.25% trypsin and penicillin mixture (double antibodies) were purchased from Israel BI. Cell Value Added Toxicity Assay Kit CCK-8 was purchased from Shanghai Biyuntian; dimethyl sulfoxide (cell-culture grade, purity > 99.7%) was purchased from Sigma, (St. Louis, MO, USA).

### 2.2. Preparation and Extraction of Ganpu Vine Tea

Process flow: fruit selection → fruit picking → fruit washing → opening the lid to dig the flesh → washing the shell and lid → drying → filling tea leaves → high temperature blanching → low temperature cooling → medium temperature drying → finished product [23,24]. Pu er tea was made in Yunan, China; vine tea was made in Hunan, China; and citrus fruit was picked on 25 July 2020 in Guangdong, China. The sensory evaluation of Ganpu vinetea was conducted by the sensory evaluate group in our team; the score of the product was above 90 (hundred-mark system).

Aqueous extract preparation: Ganpu vine tea, Ganpu tea, and vine tea were crushed over a 40 mesh sieve. The extract was formed in a boiling water bath for 30 min with the ratio of 1:20 (tea:water) and extracted from the uinsoluble solid three times. We then combined the extract and filtrated, evaporated, and concentrated the filtrate at 65 °C under reduced pressure, protecting it from light at 4 °C for later use.

Solution preparation: We weighed 26.72 mg adenosine, 26.82 mg inosine, and 0.76 mg xanthine, respectively, dissolved them in a small amount of 0.1 mol/L sodium hydroxide, and fixed the volume with PBS into a 10 mL volumetric flask to prepare a solution with concentrations of 10, 1 and 0.5 mmol/L. We then weighed or transferred a certain amount of ADA, PNP, and XOD, dissolved them with PBS, and fixed the volume into a volumetric flask to configure the solutions using concentrations of 3.2, 1.2, or 0.005 U/mL solution.

### 2.3. In Vitro Inhibition of ADA Reaction System

We referred to and slightly modified the method used by Sun et al. [25]: 0, 1, 2.5, 5, 10, 20, 50, and 100 μL of Ganpu vine tea, Ganpu tea, and vine tea aqueous extract solutions were pipetted into each centrifuge tube, before being made up to 900 μL with PBS. In total, 100 μL ADA solution was added, and after holding at 37 °C for 15 min, 100 μL adenosine solution was added to start the reaction for 30 min at 37 °C. Later on, 100 μL of 5.5 mol/L hydrochloric acid was added to each centrifuge tube to terminate the reaction. The test was repeated three times.

### 2.4. In Vitro Inhibition of PNP Reaction System

Using the preparation and procedure of reaction system referred to in Section 2.3, we replaced 100 μL ADA solution with 100 μL PNP solution, using inosine solution as substrate solution. The test was repeated three times.

### 2.5. In Vitro Inhibition of XOD Reaction System

Using the preparation and procedure of reaction system referred to in Section 2.3, we replaced 100 μL ADA solution with 100 μL XOD solution, using xanthine solution as substrate solution. The test was repeated three times.

### 2.6. Uric Acid Inhibition through Ganpu Vine Tea in HepG2 Cells 

We used CCK-8 method to detect the effect of aqueous extract on cell activity. Resuscitated HepG2 cells were added to a complete medium (89% RMPI1640 medium, 10% fetal bovine serum, and 1% double antibody) and incubated in a cell culture incubator at 37 °C, using 5% CO_2_. When the cells grew adnexal to 80% density, they were digested using 0.25% trypsin and passaged, and logarithmic growth phase cells were taken for subsequent experiments. The cells were inoculated in 96-well plates at a 15 × 10^4^ cells/mL density. In total, 100 μL were placed into each well, with the control group (no aqueous extract intervention), Ganpu vine tea group, Ganpu tea group, and vine tea group, as well as six replicate wells, in each group. After 24 h incubation, the culture solution was aspirated, and a complete fresh medium was added to the control group. The full medium of each series of concentration gradient of aqueous extract was added to the Ganpu vine tea, Ganpu tea, and vine tea groups. After 36 h of incubation, the supernatant was removed, PBS was washed three times, and complete medium was added to each well of each group. Next, 10 μL CCK-8 solution was added, and after 4 h of incubation, it was taken out and placed on an enzyme marker. Each well’s cell Optical Density (OD) values were measured at 450 m wavelength to calculate cell viability and determine the non-toxic dose of Ganpu vine tea, Ganpu tea, and vine tea to the cells.

Changes in metabolites of a uric acid metabolic pathway in the aqueous extract intervention of high uric acid cell model: According to the results of the CCK-8, the aqueous extract concentrations of Ganpu vine tea, Ganpu tea, and vine tea that did not affect cell viability were selected to intervene in the cells. Referring to the modeling method of the high uric acid cell model by Yujuan Li and slightly modified [24], the cells were inoculated at a density of 15 × 10^4^ cells/mL in a 24-well plate, with 1 mL per well for the control group, model, positive drug, Ganpu vine tea, Ganpu tea, and vine tea groups. After 24 h incubation, the supernatant was aspirated, and a complete medium was added to the control and model groups. Next a complete medium containing febuxostat at a final concentration of 100 μmol/L was added to the positive drug group; an entire medium with each series of concentrations gradient aqueous extracts was then added to the Ganpu vine tea, Ganpu tea, and vine tea groups. After 36 h incubation, 20 μL of complete medium containing 5 mmol/L adenosine was added to each group. After 3 h of cell incubation, the supernatant was taken and detected by HPLC. 

### 2.7. HPLC Analysis

1 mL of sample solution from each of the three enzyme reaction systems was taken separately, filtered through 0.45 μm microporous membrane, and analyzed via high-performance liquid chromatography (HPLC). The products of the enzyme reaction systems were then quantified via the standard external method, which was used to calculate the inhibition rate of Ganpu vine tea, Ganpu tea, and vine tea on enzymes related to uric acid synthesis.

Reaction products in the enzyme reaction system were determined via high-performance liquid chromatography, referring to the method of Yujuan Li with slight modification [25], using microsorb C18 column (4.6 mm × 150 mm, 5 μm). Mobile phase A: 20 mmol/L potassium acetate and 0.52 mmol/L sodium n-pentanesulfonate (pH = 4.0); mobile phase B: methanol. The linear elution program was as follows: 0–18 min, 100%A isocratic elution; 18–28 min, 70%A + 30%B gradient elution; 28–35 min, 100%A isocratic elution column temperature, 20 °C; flow rate, 0.75 mL/min; detection wavelength, 254 nm via dipole array tube detector; and injection volume, 20 μL.

The agilent 1260 liquid chromatography system [with quadruple pump (G1311B), autosampler (G1329B), column temperature chamber (G13416A), and dipole array tube detector (G1315D) were purchased from Agilent (Santa Clana, CA, USA). The Ultrapure water instrument preparation instrument was purchased from Shenzhen Hongsen Environmental Protection Technology Co., Ltd., Shenzhen, China. 1300 SERIES A2 Ultra-clean bench was purchased from Thermo Fisher, Waltham, MA, USA. The Inverted phase-contrast microscope was purchased from McAudi Co., Xiamen, China. The Incubator was purchased from American SHELLAB Company, Cornelius, NC, USA. The Oven was purchased from Zhongxin Medical Instruments, Co., Fuzhou, China.

### 2.8. Data Analysis

The measured data were collated using excel2016; SPSS 26 software was used to analyze the data for ANOVA. Bonferroni’s method was used to test if the groups’ variances were the same., while the Games–Hoell method was used if the conflict was not the same. The statistical results were expressed as mean ± standard deviation (χ ± SD).

We calculated the inhibition rate of enzymes related to uric acid synthesis in vitro with aqueous extracts using the following formula:

$$I=\frac{{C_{control,30}-C}_{extract,30}}{C_{control,30}-C_{control,0}}\times 100\%$$

where $C_{control,30}$ is the concentration of the product after 30 min reaction in the enzyme reaction system without aqueous extract, $C_{extract,30}$ is the concentration of the product after 30 min reaction in the enzyme reaction system with aqueous extract, and $C_{control,0}$ is the concentration of the product after 0 min reaction in the enzyme reaction system without aqueous extract. The hydrochloric acid is added immediately to abort the response.

## 3. Results

### 3.1. Results of Inhibition of Uric Acid Synthase Using Aqueous Extract

**Figure 2:**
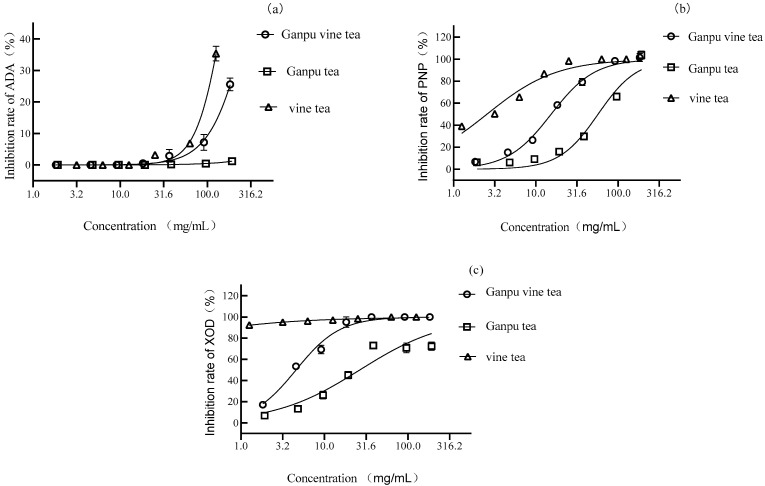
Inhibition rate of enzyme in the in vitro enzyme reaction system. (**a**) ADA; (**b**) PNP; (**c**) XOD.

In the constructed ADA, PNP, and XOD enzyme reaction systems, Ganpu vine tea, Ganpu tea, and vine tea aqueous extracts were added, respectively. It was found that Ganpu vine tea and vine tea had specific inhibitory effects on ADA (see Figure 2a), with half-inhibitory concentrations (IC_50_) of 313.10 and 156.60 mg/mL (calculated as the corresponding tea mass concentration at the time of preparation of aqueous extracts). In contrast, Gansu tea had no significant inhibitory effect. Ganpu vine tea, Ganpu tea, and vine tea inhibited PNP (see Figure 2b), with IC_50_ of 15.35, 57.13, and 2.55 mg/mL, respectively, with vine tea inhibiting PNP 6.0 and 22.4 times more than Ganpu vine tea and Ganpu tea. Vine tea inhibited XOD significantly (see Figure 2c), with an IC_50_ of 0.01 mg/mL, which was 351.8 and 2042.3 times more than the inhibitory ability of Ganpu vine tea and Ganpu tea. The results showed that the inhibitory effects of vine tea on ADA, PNP, and XOD were generally better than those of Ganpu vine tea and Ganpu tea, and the inhibitory effects on XOD were significant. The main active ingredients of vine tea are flavonoids, such as lignocaine, quercetin, etc., which achieve XOD inhibition through occupying the XOD active site or inducing changes in its molecular conformation [26,27]. The Ganpu vine tea was made through adding raw materials of vine tea, which have an enhanced ability to inhibit enzymes related to uric acid synthesis; the raw materials especially show good inhibition of XOD, which is a key enzyme for uric acid production, probably because of the increase in flavonoids and the enhanced ability to inhibit XOD, leading to a decrease in uric acid synthesis in vivo.

### 3.2. Results of Inhibition of Uric Acid Production Using Aqueous Extracts

#### 3.2.1. High Uric Acid Cell Model Construction

**Figure 3:**
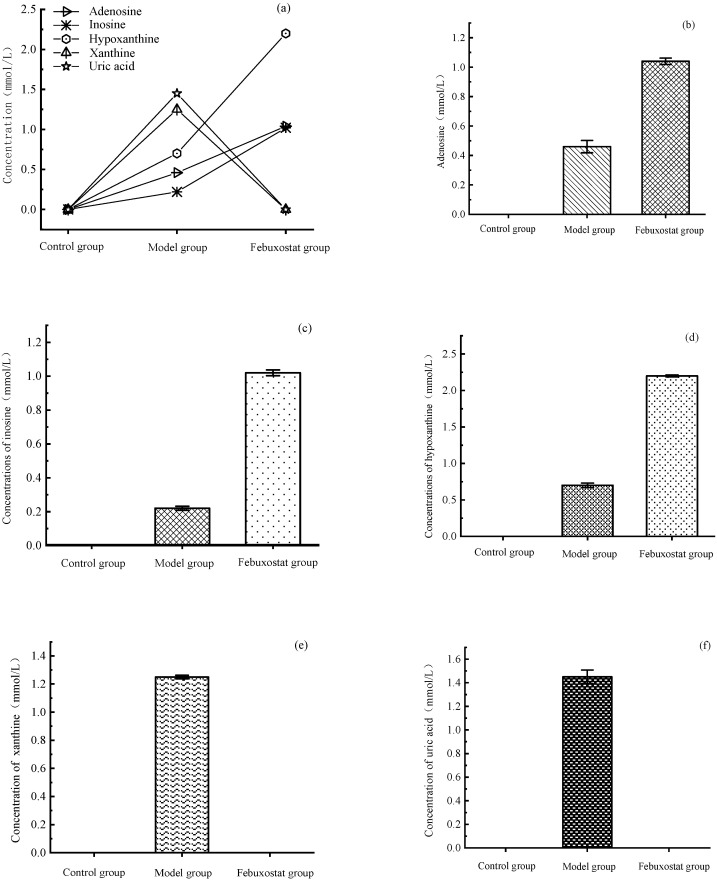
Concentrations of metabolites in HepG2 cells supernatant. (**a**) Concentrations of metabolites on the uric acid synthesis pathway in different group; (**b**) Adenosine; (**c**) Inosine; (**d**) Hypoxanthine; (**e**) Xanthine; (**f**) Uric acid.

As shown in Figure 3, after 3 h of adenosine induction, in the cell supernatant, each metabolite on the uric acid synthesis pathway was below the detection limit in the control group. The levels of adenosine, inosine, hypoxanthine, xanthine, and uric acid were significantly higher in the model group than in the control group (*p* ≤ 0.01), indicating successful modeling of the hyperuricemic cell model. The positive drug group was intervened through adding febuxostat before adenosine modeling, which inhibits both the oxidation and reduction states of XOD [6]. Therefore, XOD catalyzed the generation of xanthine from hypoxanthine. Uric acid was then inhibited, causing a decrease in xanthine and uric acid levels while allowing the accumulation of adenosine, inosine, and hypoxanthine to occur. As soon as one step of the enzymes of the uric acid synthesis pathway is inhibited, the entire uric acid synthesis pathway substances are in a state of linkage.

#### 3.2.2. Results of Cytotoxicity Assay

**Figure 4:**
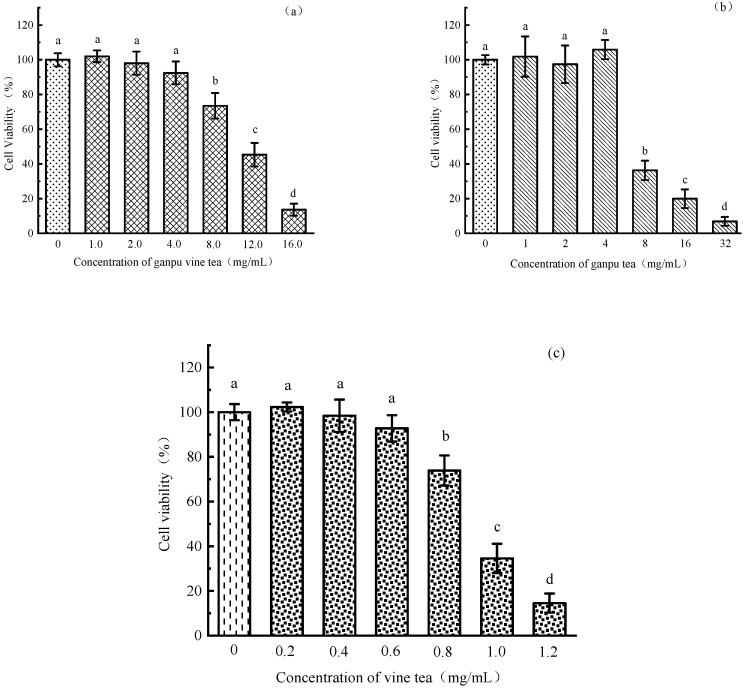
Effect of HepG2 cells viability based on different concentrations of 3 kinds of tea. (**a**) Ganpu vine tea; (**b**) Ganpu tea; (**c**) Vine tea. Notes: “a–d” indicate the different significant level.

The cellular activities of the Ganpu vine tea group, Ganpu tea group, and vine tea group were detected via the cck-8 method. It was found that a specific concentration of Ganpu vine tea, Ganpu tea, and vine tea (calculated as the corresponding tea mass concentration at the time of preparation of the aqueous extract) had no significant cytotoxicity on HepG2 compared with the control group (see Figure 4). Therefore, the concentrations of Ganpu Vine tea, Ganpu tea, and Vine tea that did not affect cell viability were selected for the intervention study.

#### 3.2.3. Aqueous Extract Intervention Cell Model Experiment for High Uric Acid

**Figure 5:**
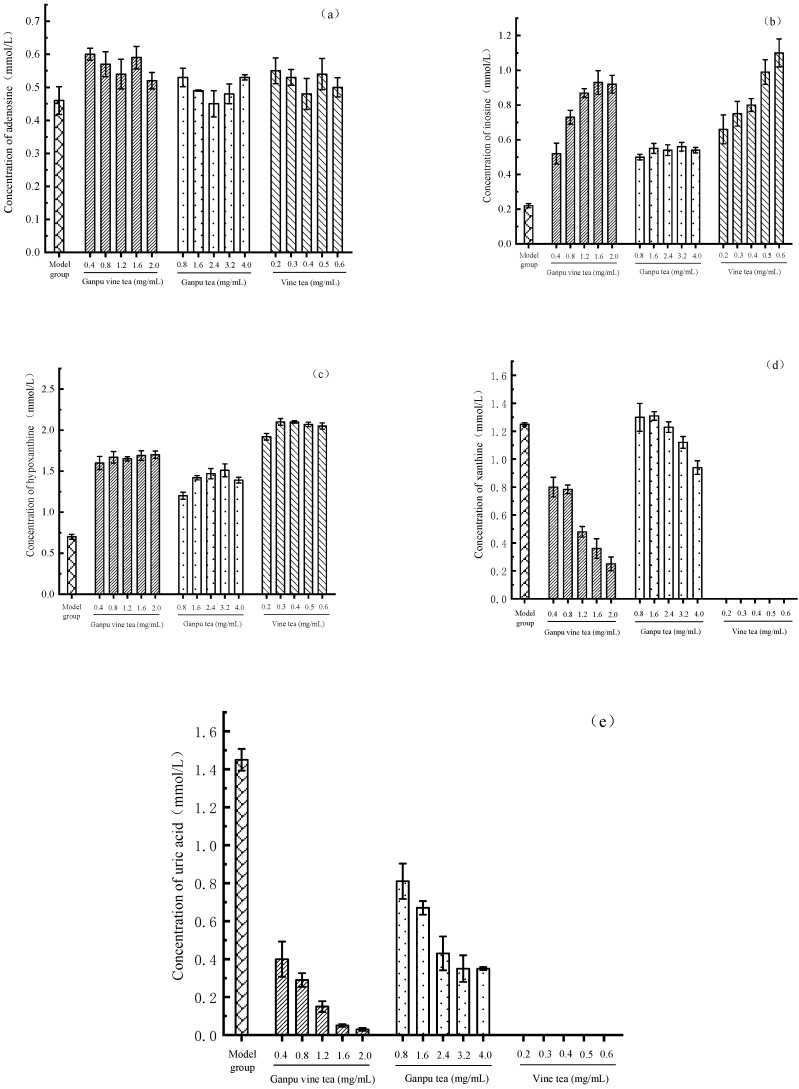
Concentrations of metabolites in supernatant of HepG2 cells incubated with different concentration of ganpu vine tea, ganpu tea, and vine tea. (**a**) Adenosine; (**b**) Inosine; (**c**) Hypoxanthine; (**d**) Xanthine; (**e**) Uric acid.

The aqueous extract intervention cell model experiment found that the adenosine content of Ganpu vine tea, Ganpu tea, and vine tea all changed little compared with the model group (see Figure 5a), inosine and hypoxanthine accumulated to different degrees compared with the model group (see Figure 5b,c), and xanthine and uric acid concentrations decreased (see Figure 5d,e). It can be seen that all tea extracts significantly reduced (*p* ≤ 0.01) uric acid production in the hyperuricemic cell model, and the best uric acid-lowering effect was achieved using vine tea. In total, 0.2 mg/mL vine tea reduced both xanthine and uric acid to 0 mmol/L. A total of 1.6 mg/mL Ganpu vine tea decreased uric acid content to 0.05 mmol/L compared to Ganpu tea at the same concentration, which reduced uric acid content in ADA. PNP was inhibited via the aqueous extract intervention, while inosine and hypoxanthine in the uric acid synthesis pathway were blocked from generating products through enzymatic reactions, causing both substrate concentrations to significantly decrease (*p* ≤ 0.01). At the same time, the aqueous extract inhibited XOD and hindered the stepwise conversion of hypoxanthine and xanthine to uric acid. This outcome suggests that the changes in the concentration of each substance during the metabolism of adenosine to uric acid in the cell model are the result of the combined action of ADA, PNP, and XOD.

## 4. Discussion

The concentration of aqueous extracts in the high uric acid cell model was much lower than in vitro inhibition of uric acid synthesis-related enzymes. Nevertheless, the combined inhibition of ADA, PNP, and XOD eventually made the aqueous extracts exhibit a good uric acid-lowering effect. In addition, because of the cellular model, the aqueous extracts altered the nucleic acid metabolic process via modulating uric acid synthesis-related enzymes to reduce uric acid content, as well as altering intracellular amino acid metabolism, lipid metabolism, energy metabolism, etc. For example, in an analysis of the metabolic intervention of Pu’er ripe tea in HUA mice, Zhao Ran [12] found that the Pu’er tea group significantly downregulated the levels of products related to energy metabolism (mannitol and erythrose-4-phosphate) compared to the model group, thus reducing the energy provided to the process of purine metabolism to produce uric acid and achieving a uric acid-lowering effect. Therefore, the aqueous extract achieves the ultimate uric acid-lowering effect through inhibiting enzymes related to uric acid synthesis and regulating multiple metabolic substances in various cell metabolic pathways. The ability to control the main target of uric acid-lowering effect uric and acid synthesis related enzymes in order of size is as follows: vine tea > Ganpu vine tea > Ganpu tea; thus, the final cell model of uric acid-reducing effect is in the following order: vine tea > Ganpu vine tea > Ganpu tea. Adding vine tea to the Ganpu tea elevates the uric acid-reducing effect of Ganpu tea; thus, the new-made Ganpu vine tea is not only improved in terms of taste, but is also superior to the Ganpu tea in terms of healthiness.

The ability of Ganpu vine tea to inhibit uric acid production is more robust than that of Ganpu tea because the raw material of vine tea is added to the production process of Ganpu vine tea. The flavonoid content of vine tea is above 400 mg/g, which is considered an important class of compounds for the hyporheic acid effect of vine tea. It can significantly reduce the uric acid content and ADA and XOD activities in the serum of HUA mouse models [28,29]. In addition, multiple components in the aqueous extract interacted to form a multi-component single-target receptor-ligand binding pattern on uric acid synthase, resulting in a weakened or enhanced ability to inhibit the target enzyme activity. Zeng Ni [30] investigated the effect of soluble sugars on the inhibition of XOD activity by flavonoids and found that fructose, sucrose, and hydrosucrose could bind to laccase, weakening its ability to inhibit XOD activity. Therefore, Gansu tea did not inhibit ADA enzyme activity strongly in the in vitro ADA inhibition assay, which may be because multiple components in the aqueous extracts interacted to reduce the active ingredients’ actual inhibitory efficacy. Xuejie Wang [31] used the molecular docking simulation technique to screen ADA target proteins with chicory small molecule compounds and found 56 compounds that inhibited ADA had flavonoid components, such as quercetin-7-O-glucosidic acid and kaempferol-7-O-glucoside. These substances were also present in Ganpu tea. Moreover, the active ingredients in aqueous extracts undergo complex biotransformation from ingestion into the human body to direct action on target cells and enzymes. Whether their bioavailability is good still needs to be studied in depth.

## 5. Conclusions

Through constructing an in vitro uric acid synthase inhibition reaction system and comparing the ability of Ganpu vine tea, Ganpu tea, and vine tea to inhibit uric acid synthase, we found that vine tea had the best ability to inhibit ADA, PNP, and XOD in vitro. The aqueous extract intervention in the high uric acid cell model found that vine tea also had the best effect in lowering uric acid. The main active ingredients in vine tea are flavonoids, and the raw material of vine tea was added in the production of Ganpu vine tea to transform its inclusion. Vine tea’s in vitro inhibition of uric acid synthase and the cellular model of uric acid lowering effect was superior to that of Ganpu tea. Ganpu vine tea uses a multicomponent, multitarget synergistic effect to achieve the result of uric acid reduction, while the mechanism of action for its practical components to inhibit uric acid production and its actual impact on the prevention and treatment of HUA need to be further studied.

## Figures and Tables

**Table 1:** Main chemical components of tested tea.

Tea Name	Constitute	Main Chemical Components
Pu er tea	Tea leaves (fermented)	Polysaccharides, catechin, tea polyphenol
Ganpu tea	Citrus shell and Pu er tea	Polysaccharides, catechin, tea polyphenol, nobiletin, hesperidin
Vine tea	Leaves of vine (dried)	Dihydromyricetin, polyphenols
Ganpu vine tea	Citrus shell, Pu er tea and vine tea	Polysaccharides, catechin, polyphenol, nobiletin, hesperidin, dihydromyricetin

## Data Availability

No new data created.
